# The Effect of a Polyester Nanofibrous Membrane with a Fibrin-Platelet Lysate Coating on Keratinocytes and Endothelial Cells in a Co-Culture System

**DOI:** 10.3390/nano11020457

**Published:** 2021-02-11

**Authors:** Andreu Blanquer, Jana Musilkova, Elena Filova, Johanka Taborska, Eduard Brynda, Tomas Riedel, Andrea Klapstova, Vera Jencova, Jana Mullerova, Eva Kuzelova Kostakova, Renata Prochazkova, Lucie Bacakova

**Affiliations:** 1 Institute of Physiology of the Czech Academy of Sciences, Videnska 1083, 142 20 Prague 4, Czech Republic; jana.musilkova@fgu.cas.cz (J.M.); elena.filova@fgu.cas.cz (E.F.); Lucie.Bacakova@fgu.cas.cz (L.B.); 2 Institute of Macromolecular Chemistry of the Czech Academy of Sciences, Heyrovskeho nam. 2, 162 06 Prague 6, Czech Republic; taborska@imc.cas.cz (J.T.); brynda@imc.cas.cz (E.B.); riedel@imc.cas.cz (T.R.); 3 Faculty of Textile Engineering, Technical University of Liberec, Studentska 1402/2, 461 17 Liberec 1, Czech Republic; andrea.klapstova@tul.cz; 4 Faculty of Science, Humanities and Education, Technical University of Liberec, Studentska 1402/2, 461 17 Liberec 1, Czech Republic; vera.jencova@tul.cz (V.J.); Jana.mullerova@tul.cz (J.M.); Eva.kostakova@tul.cz (E.K.K.); 5 Institute of Nanomaterials, Advanced Technologies and Innovation, Bendlova 1409/7, 460 01 Liberec 1, Czech Republic; 6 Faculty of Health, Technical University of Liberec, Studentska 1402/2, 461 17 Liberec 1, Czech Republic; renata.prochazkova@nemlib.cz; 7 Regional Hospital Liberec, Husova 357/28, 460 01 Liberec 1, Czech Republic

**Keywords:** electrospun nanofibre, skin wound healing, keratinocytes, platelet lysate, fibrin, endothelial cells, in vitro co-culture system

## Abstract

Chronic wounds affect millions of patients worldwide, and it is estimated that this number will increase steadily in the future due to population ageing. The research of new therapeutic approaches to wound healing includes the development of nanofibrous meshes and the use of platelet lysate (PL) to stimulate skin regeneration. This study considers a combination of a degradable electrospun nanofibrous blend of poly(L-lactide-*co*-ε-caprolactone) and poly(ε-caprolactone) (PLCL/PCL) membranes (NF) and fibrin loaded with various concentrations of PL aimed at the development of bioactive skin wound healing dressings. The cytocompatibility of the NF membranes, as well as the effect of PL, was evaluated in both monocultures and co-cultures of human keratinocytes and human endothelial cells. We determined that the keratinocytes were able to adhere on all the membranes, and their increased proliferation and differentiation was observed on the membranes that contained fibrin with at least 50% of PL (Fbg + PL) after 14 days. With respect to the co-culture experiments, the membranes with fibrin with 20% of PL were observed to enhance the metabolic activity of endothelial cells and their migration, and the proliferation and differentiation of keratinocytes. The results suggest that the newly developed NF combined with fibrin and PL, described in the study, provides a promising dressing for chronic wound healing purposes.

## 1. Introduction

The healing of skin wounds is a process that involves several types of cells including keratinocytes, fibroblasts, endothelial cells and immune cells. Various signalling molecules serve to regulate the cellular response and the dynamic remodelling of the extracellular matrix in the various wound healing phases. The ability of cells to respond to injury via migration, proliferation and the production of cytokines, enzymes and other bioactive molecules is critical in terms of initiating the wound healing process. However, under certain circumstances, the healing process is retarded or ceases completely, thus leading to the emergence of chronic wounds including pressure ulcers, venous leg ulcers, arterial ulcers, neurotrophic ulcers and foot ulcers in persons with diabetes [1]. The prevalence of chronic wounds increases with the occurrence of vascular diseases and diabetes mellitus, as well as systemic factors such as advanced age. Moreover, millions of surgical wounds that have the potential to develop into chronic wounds occur as part of routine medical care. Indeed, it is estimated that 1–2% of the population experience chronic wounds during their lifetime [2]. Thus, it is essential that new efficient therapeutic approaches be developed aimed at enhancing the wound healing process.

Nanofibrous membranes (NF) comprise promising materials for the regeneration of damaged skin since they mimic the fibrous component of the extracellular matrix (ECM) and are able to easily adapt to the shape of the wound. In addition, nanofibrous meshes are capable of acting as a protective barrier to the penetration of microorganisms into the wound and allow for the exchange of gases and the absorption of the wound exudate [3]. Various types of synthetic degradable polymers have already been used as scaffolds for skin tissue engineering purposes. Polycaprolactone (PCL) comprises one of the most frequently employed polymers in this respect due to its high degree of solubility in a variety of solvents, as well as its tuneable mechanical properties and good biocompatibility properties. However, PCL is highly hydrophobic, which reduces its ability to support cell adhesion and growth. In order to mitigate this problem, PCL can be combined with polylactic acid (PLA) so as to obtain a poly(L-lactide-*co*-ε-caprolactone) (PLCL) copolymer. The PLCL copolymer evinces an enhanced degradation rate and a higher degree of wettability than does PCL [4], both of which are favourable properties with respect to biomedical applications.

In addition, polymeric nanofibrous scaffolds can be combined with bioactive substances in order to enhance skin healing and regeneration. Wound healing is a dynamic and complex cascade of events that involves several cell types and that is governed by multiple cytokines and growth factors [5]. In some chronic wound cases, the lack of availability of growth factors negatively affects the healing cascade, and several authors have suggested the therapeutic use of these factors so as to accelerate tissue healing [6]. The potential beneficial effects of a wide range of growth factors have already been extensively investigated with concern to keratinocytes and other cell types involved in the skin regeneration process such as endothelial cells, fibroblasts and epidermal stem cells [7,8,9,10]. These factors include platelet-derived growth factor (PDGF), transforming growth factor-beta (TGF-β), platelet-derived epidermal growth factor (PDEGF), epidermal growth factor (EGF), vascular endothelial growth factor (VEGF), fibroblast growth factor (FGF), insulin-like growth factor (IGF) and tumour necrosis factor-alpha (TNF-α) [11]. In this regard, platelet derivatives have been studied as sources of a high number of bioactive compounds released during blood coagulation. The main components of these derivatives include coagulation factors, vasoactive factors and the growth factors mentioned above. Several types of platelet derivatives have been described, including platelet-rich plasma, platelet gel and platelet lysate (PL), with clear evidence of their therapeutic application [12]. Of the various types of platelet derivatives, the PL effect has been widely analysed in both in vitro and in vivo studies, and the results demonstrated that PL accelerates the proliferation of fibroblasts and endothelial cells [5,13]. In addition, PL was found to enhance the proliferation and migration of keratinocytes [14,15].

Recently, by pooling the benefits of nanofibrous meshes and bioactive molecules, several authors have combined various types of polymers with growth factors, plant extracts or antibiotics as potential dressings for chronic wounds and subsequent skin regeneration [16,17]. We combined the advantages of electrospun PLCL/PCL membranes and PL biomolecules in the form of a wound dressing that aimed to enhance the proliferation and differentiation of skin cells. The PLCL/PCL nanofibres were assembled with a fine fibrin mesh containing the PL. We then investigated the cytocompatibility of these novel potential composite nanofibrous skin wound dressings using keratinocytes. The initial adhesion, morphology, proliferation and differentiation of these cells were analysed using various concentrations of PL (1%, 5%, 10%, 20%, 50% and 100%) added to the fibrin coatings of the NF membranes, as well as via two control samples (pure membranes and membranes assembled as fibrin meshes without PL). In addition to this conventional cell monoculture system, keratinocytes were co-cultured on nanofibrous meshes with endothelial cells grown on the underlying bottoms of polystyrene wells, which was followed by the analysis of the behaviour of both cell types. This system provided for a more realistic model of the situation in wounds in vivo, where both the paracrine production of various compounds and cell–cell interactions contribute to the wound healing process.

## 2. Materials and Methods

### 2.1. The Fabrication and Characterization of the PLCL/PCL Nanofibre Membrane (NF)

A blend of poly(L-lactide-*co*-ɛ-caprolactone) (PLCL) and poly(ε-caprolactone) (PCL) nanofibres was deposited on a sheet of supporting polypropylene fabric by means of the DC needleless electrospinning technique (Nanospider 1WS500U, Elmarco, Liberec, Czech Republic). The electrospinning solution contained a mixture of 5% (*w*/*w*) PLCL (Purasorb PLC 7015, Corbion, Amsterdam, Netherlands) and 5% (*w*/*w*) PCL (PCL; Mn 80,000 g/mol; Merck, Darmstadt, Germany) in a chloroform/ethanol/acetic acid solution (8:1:1). PLCL spinnability via needleless electrospinning has been described previously [18], as well as the spinnability of PCL [19]. The combination of these two polymers demonstrated similar process parameters, which were subsequently optimized with respect to optimal spinnability. The electrospinning was performed at a humidity of 50% and a temperature of 22 °C ensured by a precisely controlled air conditioning system (NS AC150, Elmarco, Liberec, Czech Republic). Voltages of −10 kV and +40 kV were applied to the collector and the string respectively. The string (electrode) distance was 190 mm from the collector and the rewinding speed was set at 18 mm/min. Prior to the characterization of, and further modifications to, the prepared PLCL/PCL nanofibre membrane, the membrane was gently removed from the polypropylene fabric and cut into samples of the desired dimensions. The upper and bottom surfaces of the NF samples were sputter coated with 7 nm of gold (Quorum Q50ES; Quorum Technologies, Laughton, UK), and micrographs of the surfaces were taken using a Vega Tescan 3 microscope (Tescan, Brno, Czech Republic) at 10–20 kV. The cells were in all cases seeded on the upper NF surface. The distribution of the PLCL/PCL fibre diameters was evaluated from the micrographs of 200 randomly measured fibres using NIS Elements software. The density of the membrane was 22 ± 4 g/m^2^.

### 2.2. Platelet Lysate (PL) Preparation

A platelet concentrate (PRS) was obtained via the centrifuge separation of platelets from human buffy coats (BC) of the same blood group. The BC were prepared from 450 ± 45 mL whole blood collected in quadruple bags (CompoFlow 4F, Fresenius Kabi AG, Bad Homburg, Germany), treated with an anticoagulant citrate phosphate dextrose (CPD) solution at a ratio of 1:7 and subsequently subjected to hard-spin centrifugation at 3250 rpm for 14 min; this was followed by processing in an automated blood component separator (Compomat G5, Fresenius Kabi AG, Bad Homburg, Germany). The BC were then stored overnight at 22 °C with constant agitation. Four BC together with a platelet additive solution containing 70% of Composol (Fresenius Kabi AG, Bad Homburg, Germany) and 30% of blood plasma were coupled to a pooling set (Compostop Flex 3F T&B, Fresenius Kabi AG, Bad Homburg, Germany) through a sterile connection device (TSCD ^®^ II, Terumo BCT) and pooled in the bags. Low-speed differential centrifugation was applied at 1150 rpm for 8 min so as to separate the platelet concentrate from the red blood cells and leukocytes. Following filtration, the resulting content of leucocytes was determined at <1 × 10^6^ (Compomat G5, Fresenius Kabi AG, Bad Homburg, Germany). The PRS were stored at 22–24 °C with constant agitation. The PL were then prepared using the freeze–thaw method. A bag of PRS was frozen (−80 °C) and thawed (37 °C) so as to disrupt the platelets. The PL were subsequently centrifuged in order to dispose of the cellular debris, and aliquots of the solution were stored at −80 °C. Since the PRS concentrations in the bags varied within an interval of 600–900 × 10^6^ platelets per 1 mL, the PL prepared from the various PRS bags were normalized via dilution with Tris buffer (TB) prior to the experiment so as to correspond to a pre-selected standard PRS concentration of 600 × 10^6^ platelets per 1 mL.

### 2.3. Quantification of the Growth Factors in the PL

Concentrations of the selected growth factors (FGF, VEGF and PDGF-BB) in the PL aliquots were measured by means of an enzyme-linked immunosorbent assay (ELISA) following the instructions in the manufacturer’s manual. The PDGF-BB Human ELISA Kit (Cat. No. BMS2071), FGF Human ELISA Kit (Cat. No KHG0021) and VEGF Human ELISA Kit (Cat. No. KHG0111) were purchased from Thermo Fisher Scientific, Waltham, MA, USA.

### 2.4. Coating of the NF with Fibrin and PL Assemblies

**Figure 1:**
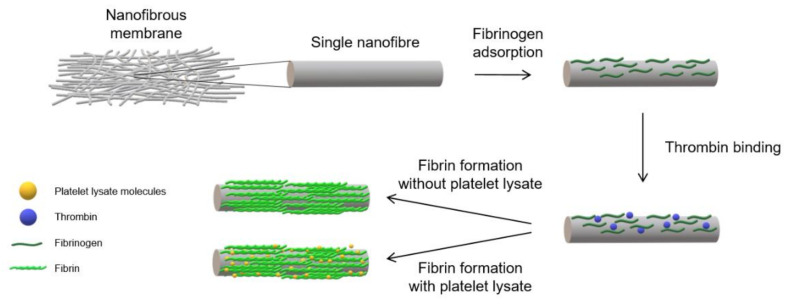
Schematic illustration of the single nanofibre coating procedure with fibrin and platelet lysate.

Circular samples with areas of 1.9 cm^2^ were cut from the NF, placed in 70% ethanol, exposed to UV light for 30 min and rinsed in sterile deionized water. The water was changed each day for 5 days in order to remove the solvent residua. Prior to coating, the samples were transferred to the wells of 24-well plates with sterile Tris buffer (TB)—0.05 M Trizma (Sigma-Aldrich, Merck, Darmstadt, Germany), 0.01 M NaCl and 2 mM CaCl_2_ (both from Lach-Ner, Neratovice, Czech Republic). The samples were then pre-modified via successive incubation with 5 µg/mL of fibrinogen (Sigma-Aldrich, Merck, Darmstadt, Germany) in TB overnight at 4 °C. The samples were rinsed on the following day with TB and incubated with a 2 U/mL solution of thrombin (Sigma-Aldrich, Merck, Darmstadt, Germany) in TB for 30 min at room temperature (RT). The final step comprised the further modification of the pre-modified NF with various coatings. The NF coated with a fibrin mesh (NF0) was prepared by means of incubating the pre-modified NF with a solution of 200 µg/mL of fibrinogen and 0.5 U/mL of antithrombin III (ATIII, Chromogenix, London, UK) in TB for 2 h at RT. A schematic illustration of the developed coating procedure is shown in Figure 1. The NF coated with assemblies containing fibrin and various concentrations of PL were prepared by means of incubating NF with solutions containing Fbg + 1% PL, Fbg + 5% PL, Fbg + 10% PL, Fbg + 20% PL, Fbg + 50% PL and 100% PL for 2 h at RT, and were labelled as NF1, NF5, NF10, NF20, NF50 and NF100, respectively. The solutions were prepared by mixing selected volumes of PL with a solution containing 200 µg/mL of fibrinogen and 0.25 U/mL of ATIII in TB, so that the resulting solutions contained 1%, 5%, 10%, 20% and 50% of PL in the total volume (*v/v*). A sample coated only with PL (NF100) was prepared via the incubation of NF with 100% PL. The samples were washed with TB following each stage and finally washed with sterile PBS.

### 2.5. Coating Characterization

The amount of proteins in the coatings was quantified via the dual application of a bicinchoninic acid (BCA) protein assay kit (ThermoFisher Scientific, Waltham, MA, USA). The fibrin in the coatings was degraded via the incubation of a coated NF sample with a plasmin (Roche, Sigma-Aldrich, Merck, Darmstadt, Germany) solution (0.01 U/mL in PBS) overnight at 37 °C, which was followed by the measurement of the concentration of the released proteins in the resulting solution by means of BCA. The concentration of residual proteins that remained in the NF was determined via the application of BCA to the sample.

The amounts of FGF, VEGF and PDGF-BB in a solution obtained via the plasmin degradation of the NF20 coating were determined using the respective ELISA kits.

The proteins in the coating were fluorescence stained using fluorescamin (Acros Organics, Geel, Belgium) and the structure of the coating in the surface region of the NF was observed using an Olympus IX83 confocal microscope (Olympus, Tokyo, Japan).

The porosity and pore size of NF membranes were measured automatically using NIS Element software (Nikon, Melville, NY, USA). Data were obtained from a total of 10 images; PLCL/PCL nanofibrous membranes were compared before and after coating.

### 2.6. Infrared Spectroscopy (FT-IR) and Raman Spectroscopy

The chemical composition of materials was investigated by attenuated total reflection Fourier transform infrared spectroscopy (ATR-FTIR) using a Fourier transform spectrometer (Nicolet iZ10, ThermoFisher Scientific, Waltham, MA, USA) at room temperature. The samples were placed on the ATR diamond crystal for analysis and spectrum analysis was performed in the infrared region in the range of 400–4000 cm^−1^ and spectral resolution of 4 cm^−1^. Coated NF membranes were dried out by lyophilization from Milli-Q water. Raman spectroscopy analysis was performed on a single fibre using Raman DXR microscope (ThermoFisher Scientific, Waltham, MA, USA) using 532 nm laser wavelength.

### 2.7. Wetting

A droplet of HLPC water (10 µL) was deposited on surface of NF and coated NF dried out by lyophilization from Milli-Q water. The time between the deposition and complete penetration of the drop into the membrane was measured.

### 2.8. Cell Culture

Human HaCaT keratinocytes (CLS Cell Lines Services, Cat. No. 300493, Eppelheim, Germany) were cultured in Dulbecco’s Modified Eagle’s Medium (DMEM; ThermoFisher Scientific, Waltham, MA, USA) with 10% foetal bovine serum (FBS; ThermoFisher Scientific, Waltham, MA, USA) under standard conditions (37 °C and 5% CO_2_). The experiments with the NF membranes were performed using DMEM with 2% FBS. Primary human saphenous vein endothelial cells (HSVECs; Provitro, Cat. No. 1210121, passages 3–4) were cultured in Endothelial Growth Medium (EGM-2) with supplements (PromoCell, Cat. No. C-22111, Heidelberg, Germany). With respect to the experiments with the NF membranes, the HSVECs were cultured in low-supplemented EGM-2 containing FBS, heparin, ascorbic acid and hydrocortisone, without the presence of a growth factor.

### 2.9. Co-Culture System

**Figure 2:**
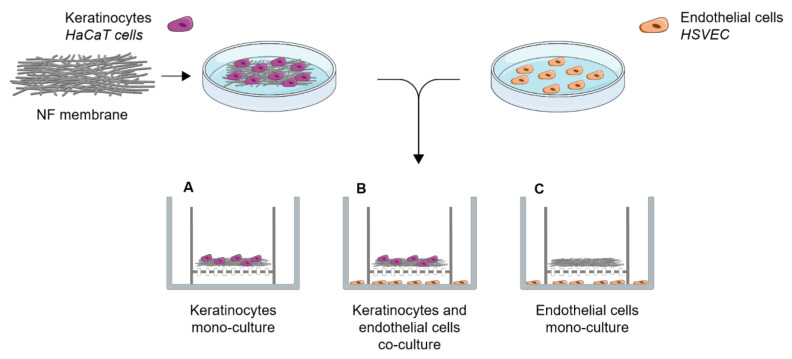
Schematic illustration of the co-culture system used in the experiments with the HaCaT cells and the human saphenous vein endothelial cells.

Human HaCaT keratinocytes and HSVECs (Provitro, Berlin, Germany) were co-cultured in vitro using cell culture inserts. A total of 7000 HaCaT cells were seeded onto the NF membranes in 0.5 mL of DMEM with 2% FBS and, after the 3 h required for cell adhesion, the membranes were placed in cell culture inserts with a pore size of 0.4 μm (Cat. No. 140652, ThermoFisher Scientific, Waltham, MA, USA). In addition, 20,000 HSVECs were seeded on 12-well plate (Techno Plastic Products, Trasadingen, Switzerland). The inserts bearing the NF membranes with keratinocytes were then placed on top of the wells with the HSVECs, as illustrated in Figure 2. At the beginning of the co-culturing process, 0.5 mL of the medium used for the HaCaT cells was added to the inserts and 1 mL of the HSVECs medium was added to the wells. The cells were co-cultured for 7 and 14 days. Both media were replaced twice, i.e., after 7 and 11 days, in the case of the 14-day experiment. The HaCaT cells were cultured in DMEM with 2% FBS. Low-supplemented EGM-2 containing FBS, heparin, ascorbic acid and hydrocortisone was used for the HSVECs. In parallel, monocultures of each of the cell types were established in the same culture setting and the same growth medium (Figure 2).

### 2.10. Cell Viability/Metabolic Activity

CellTiter 96^®^ AQueous One Solution Cell Proliferation Assay (MTS, Promega Corporation, Madison, WI, USA) was used to determine the cell viability/metabolic activity, which is directly related to the cell number. With respect to the monoculture experiments, HaCaT cells were seeded on NF membranes emplaced on a 24-well plate (Techno Plastic Products, Trasadingen, Switzerland) at a density of 7000 cells per well. Concerning the co-culture experiments, HaCaT cells were seeded on NF membranes, which were emplaced in inserts with a pore size of 0.4 µm (ThermoFisher Scientific, Waltham, MA, USA) at a density of 7000 cells per well, and 20,000 HSVECs were seeded on the PS surface of 12-well companion plates (Techno Plastic Products, Trasadingen, Switzerland). The viability/metabolic activity of the cells was measured at different time points according to the type of experiment. The monocultures of the HaCaT cells were analysed after 1, 3, 7 and 14 days in the culture, whereas the HaCaT cells in the co-cultures with HSVECs were analysed after 7 and 14 days of cultivation. The NF membranes with the HaCaT cells were transferred to fresh 24-well plates for the MTS analysis. The samples were incubated with DMEM supplemented with 2% of FBS and 100 µL of MTS for 1 h, and the absorbance was measured using a VersaMax ELISA Microplate Reader spectrophotometer (Molecular Devices Corporation, Sunnyvale, CA, USA) at a wavelength of 490 nm, and normalized per well.

### 2.11. Cell Adhesion, Morphology and Spreading Area

Following the performance of the MTS assay, the cells were rinsed with PBS and fixed with 4% paraformaldehyde in PBS for 30 min. The initial cell adhesion and morphology after 1 day in the culture were analysed via the staining of the cells with phalloidin so as to allow for the visualization of the actin filaments. The samples were washed with PBS and then incubated with TRITC-conjugated phalloidin (100 ng/mL in PBS, Sigma-Aldrich, Merck, Darmstadt, Germany, Cat. No. P1951) and Hoechst 33,258 (5 µg/mL in PBS, Sigma-Aldrich, Merck, Darmstadt, Germany, Cat. No. 861405) for 20 min at room temperature (RT). Images of the adherent cells were captured under an IX-50 microscope equipped with a DP 70 digital camera (both from Olympus, Tokyo, Japan) and a confocal laser scanning microscope (CLSM, Leica SPE, Wetzlar, Germany). The cell numbers were determined by counting the cell nuclei using ImageJ software (version 1.53, NIH, Rockville Pike, MD, USA). In addition, the size of the spreading area of the cells that had adhered to the membranes was measured from the same images using ImageJ software. At least 12 randomly taken microphotographs were evaluated for each analysis.

### 2.12. Quantitative Real-Time PCR (qPCR) of the Keratinocyte Differentiation

A qPCR analysis was performed so as to determine the expression profile of the six genes involved in the differentiation and maintenance of the mature phenotype of the human keratinocytes. HaCaT cells were grown for 7 and 14 days in the cell monocultures and co-culture system described above. Three different NF membranes (NF0, NF20 and NF50) and pure nanofibres (control sample) were analysed. The cells were rinsed with PBS and the membrane placed in a 1.5 mL tube filled with RNA lysis solution enriched with mercaptoethanol (1%). The total RNA was extracted from the cell cultures using a Total RNA Purification Micro Kit (Norgen Biotek Corp., Thorold, ON, Canada) according to the manufacturer’s protocol. The RNA concentration was measured using a Nanodrop spectrophotometer (ThermoFisher Scientific, Waltham, MA, USA). Reverse transcription was then performed on 1.5 µg of the total RNA using an Omniscript Reverse Transcription Kit (Qiagen, Venlo, Netherlands; Cat. No. 205113) and random hexamers (New England Biolabs, Inc., Ipswich, MA, USA), and conducted according to the manufacturer’s protocol in a Thermocycler (Biometra, Gottingen, Germany).

The mRNA levels of the specific genes were quantified employing quantitative real time 5xHOT FIREPol Probe Mix Plus (ROX; Solis BioDyne, Tartu, Estonia) and TaqMan Gene Expression Assays (ThermoFisher Scientific, Waltham, MA, USA; Cat. No. 4331182) labelled with a FAM reporter dye specific to human cytokeratin 1 (KRT1, Hs00196158_m1), cytokeratin 5 (KRT5, Hs00361185_m1), cytokeratin 10 (KRT10, Hs00166289_m1), cytokeratin 14 (KRT14, Hs00265033_m1), involucrin (IVL, Hs00902520_m1) and filagrin (FLG, Hs00856927_g1). Beta_2_ microglobulin (B2M, Hs00187841_m1) was used as the reference gene. The experimental groups of samples (i.e., the pure and modified NF membranes) were compared via three independent experiments performed in duplicates. The final reaction volume was 20 μL of the solution. The mRNA expression level was determined using the Viia™ 7 Real-time PCR System (Applied Biosystems™; ThermoFisher Scientific, Waltham, MA, USA) on a 96-well optical reaction plate. Expression values were obtained from the Ct numbers. The relative gene expression was calculated at 2^−ΔCt^.

### 2.13. Immunodetection of the Keratinocytes and the Endothelial Cell Differentiation

The development of intermediate filaments in the HaCaT cells was evaluated via the immunodetection of cytokeratin 14, associated with the proliferative stage, and cytokeratin 10, associated with the differentiation stage of the epidermal layers. The cells were fixed in 4% paraformaldehyde in PBS for 15 min at RT, permeabilized with 1% of BSA in PBS containing 0.1% Triton X-100 for 20 min, and treated with 1% of Tween for 20 min at RT. The samples were then incubated with primary antibodies, i.e., with a rabbit monoclonal anti-cytokeratin 10 antibody (1:400; Abcam, Cambridge, UK; Cat. No. ab76318), overnight at 4 °C, washed in PBS and subsequently incubated with a mouse monoclonal anti-cytokeratin 14 antibody (1:400; Abcam, Cambridge, UK; Cat. No. ab7800) overnight at 4 °C. After being rinsed twice with PBS, the samples were incubated with secondary antibodies, i.e., with Alexa Fluor 488-conjugated goat anti-rabbit antibody (1:400; ThermoFisher Scientific, Waltham, MA, USA; Cat. No. A11070) for 1 h, rinsed in PBS and then incubated for a further 1 h with an Alexa Fluor 546-conjugated goat anti-mouse antibody (1:400; Thermo Fisher Scientific, Waltham, MA, USA; Cat. No. A11003) and diluted in PBS together with Hoechst 33,258 dye (5 µg/mL) for the staining of the cell nuclei. Images from randomly selected regions were captured using an Olympus IX-50 epifluorescence microscope equipped with a DP 70 digital camera (both from Olympus, Tokyo, Japan). Additional images were captured using CLSM (Leica TCS SP8, Wetzlar, Germany). Ten microphotographs were analysed using ImageJ software in order to measure the area with differentiated cells positive for cytokeratin 10, and the results were shown as percentages of the surface area covered with the differentiated cells.

The HSVEC differentiation was analysed by means of the immunostaining of the von Willebrand factor. Following the same cell fixation and permeabilization treatment, the samples were incubated with a rabbit anti-von Willebrand primary antibody (1:400, Sigma-Aldrich, Merk, Darmstadt, Germany, Cat. No. F3520) overnight at 4 °C. After the samples had been rinsed twice with PBS, they were incubated with an Alexa Fluor 488-conjugated goat anti-rabbit secondary antibody (1:400).

### 2.14. Endothelial Transmigration Assay

The migration of the endothelial cells towards the NF membranes through 8 µm diameter pores was analysed using a transmigration assay setup. The membranes were placed on 24-well plates with a low-supplemented EGM-2 medium. FluoroBlok cell culture inserts (Corning, New York, NY, USA) were placed in each well and 20,000 HSVEC were seeded in the inserts. After 4 h in the cultures, the inserts were washed with PBS and fixed with 4% paraformaldehyde in PBS for 15 min at RT. The cells were then stained with a combination of the Texas Red C_2_-maleimide cell membrane dye (1.7 µg/mL in PBS, ThermoFisher Scientific, Waltham, MA, USA) and the Hoechst 33,258 nuclear dye (5 µg/mL of PBS) for 1 h at RT. Images of the cells that crossed the insert membrane were captured under an Olympus IX-71 epifluorescence microscope equipped with a DP80 digital camera (both from Olympus, Tokyo, Japan).

### 2.15. Statistical Analysis

The quantitative data were presented as the mean with the standard deviation. Statistical comparisons were performed using the one-way analysis of variance (ANOVA) with the Student–Newman-Keuls multiple comparisons test. In all cases, the analysis was performed using SigmaStat 4.0 (Systat Software Inc., San Jose, CA, USA), and a *p* value of *p* ≤ 0.05 was considered statistically significant.

## 3. Results

### 3.1. Growth Factor Quantification in the PL

The concentrations of FGF, VEGF and PDGF-BB were determined by means of ELISA kits in a stock solution of the platelet lysate. The detected concentration of the PDGF-BB was 16.4 ± 0.7 ng/mL. The concentrations of FGF and VEGF were approximately one order lower, i.e., 62.9 ± 5 pg/mL for the FGF and 51.2 ± 0.7 pg/mL for the VEGF. The initial low concentrations of FGF and VEGF in the PL stock solution precluded the quantification of these growth factors in the fibrin coatings.

### 3.2. Characterization of the NF

**Figure 3:**
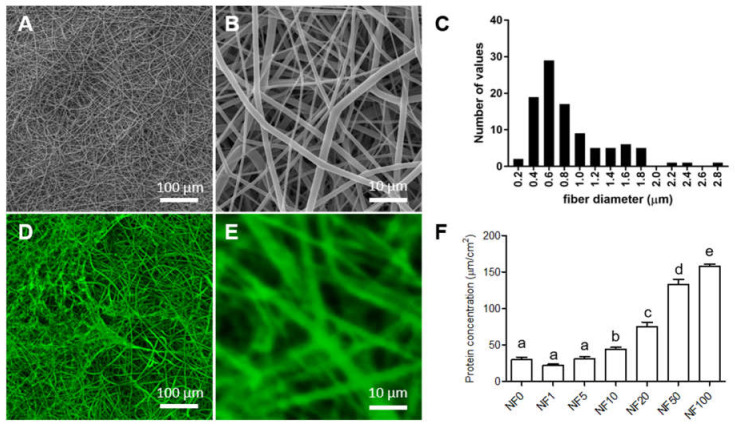
SEM images of the upper surface of a poly(L-lactide-*co*-ε-caprolactone)/poly(ε-caprolactone) (PLCL/PCL) nanofibrous membranes (**A**,**B**). Histogram of the PLCL/PCL fibre diameter distribution on the upper nanofibrous membranes’ (NF) surface (**C**). Confocal laser scanning microscope (CLSM) images measured in phosphate-buffered solution of the fluorescence-stained proteins on the upper surface of the NF modified from a solution containing Fbg and 50% of platelet lysate (PL) (NF50) (**D**,**E**). The proteins were stained with fluorescamin (green). The protein concentrations in the NF0, NF1, NF5, NF10, NF20, NF50, and NF100 prepared from solutions containing Fbg and 0%, 1%, 5%, 10%, 20%, 50% and 100% of PL (**F**). The superscript letters above the columns denote significant differences between the values that do not share the same superscript (*p* < 0.05).

The measurement of the fibre diameters in the SEM images (Figure 3A,B) revealed that the electrospun PLCL/PCL meshes contained fibres with a wide range of diameters from approx. 200 nm to 2.8 μm, most of which were in the range of 400 to 800 nm (Figure 3C). Therefore, these fibres comprised submicron-/micron-scale fibres rather than nanofibres. Fibres produced via electrospinning are commonly referred to as nanofibres, including those in this study, in which the PLCL/PCL meshes are referred to as nanofibrous meshes (NF).

The bottom surface of the NF was found to be coarser, which was probably due to its previous contact with the relatively coarse supporting polypropylene fabric that was used for the deposition of the nanofibres. The NF was modified using a specific coating procedure published previously [20,21]. This procedure, as described in paragraph 2.4 (Figure 1), included (1) the adsorption of fibrinogen on the surface of the individual nanofibres, (2) the binding of thrombin to the adsorbed fibrinogen and (3) the coating of nanofibres by a fibrin mesh formed from Fbg present in the solution used in the final stage of the coating procedure. The transformation of Fbg to fibrin was catalysed by thrombin immobilized on the nanofibres. The solutions were prepared by means of mixing a solution of Fbg in TB with various volumes of PL containing Fbg and other proteins from blood plasma and platelets.

The CLSM images measured in PBS (Figure 3D,E) show fluorescence-stained proteins (green) on the upper surface of the NF modified from a solution containing Fbg and 50% PL (NF50). The images indicate that fibrin containing other proteins from the PL was formed mainly on the surfaces of individual PLCL/PCL nanofibres. The same structures were observed in SEM images of lyophilized NF0 and NF50 membranes (Supplementary Material Figure S1). The porosity of the NF membranes before coating was 42 ± 8% with an average pore size of 10.37 ± 2.62 μm^2^ (mean ± confidence interval). The coated membranes showed porosity values of 40 ± 9% with an average pore size of 11.97 ± 2.87 μm^2^. The coating with fibrin and PL did not change the nanofibrous structure or the porosity of the NF membrane. Moreover, the CLSM images showed that the coating was stable for at least one week in PBS (Supplementary Material Figure S2).

The total amounts of proteins per 1 cm^2^ of NF coated from the fibrinogen solutions containing various PL concentrations (shown in Figure 3F) consist of fibrin and proteins from the blood plasma present in the PL. The amounts were determined via four independent experiments. The enhanced volume of PL in the fibrinogen solution acted to increase the content of protein attached to the NF. The PL that contained coatings was reinforced by the formation of a fibrin mesh from the fibrinogen present both in the fibrinogen solution and the PL plasma.

The concentration of PDGF-BB in NF20 was determined at 16.7 pg/cm^2^, whereas the concentrations of FGF and VEGF were under the detection limit of the ELISA kits of 15.6 pg/mL used for the FGF and 5 pg/mL used for the VEGF.

**Figure 4:**
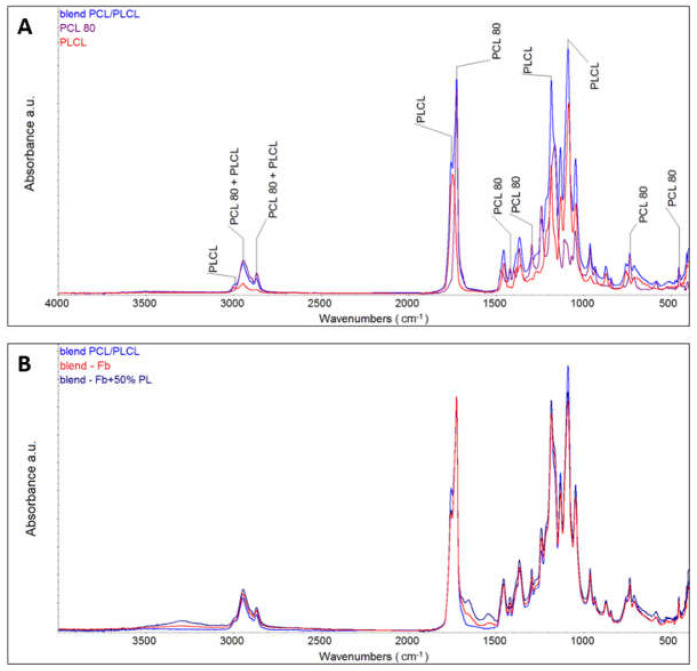
Attenuated total reflection Fourier transform infrared spectroscopy (ATR-FTIR) analysis of the PLCL/PCL and the spectra of pure PCL and pure PLCL (**A**), and lyophilized PLCL/PCL nanofibrous membranes coated with fibrin (blend-Fb) and fibrin with 50% of platelet lysate (**B**).

The chemical composition of prepared PLCL/PCL material and fibrin coated material was evaluated by FTIR analysis. The spectrum of PLCL/PCL presented characteristic peaks of PCL and PLCL (Figure 4A) at 3000 cm^−1^ (C–H stretching, PLCL), 1750 cm^−1^ (C=O stretching, PLCL), 1726 cm^−1^ (C=O stretching, PCL), and 1460 cm^−1^ (C-H deformation, PCL), 1423 cm^−1^ (C–H deformation, PCL), 1495 cm^−1^ and 1240 cm^−1^ (CH2–CO–, PLCL), 1084 cm^−1^ (C–O–C stretching, PCL), 1163 cm^−1^ (C–CO–C stretching, PCL). Amide bonds within the fibrin (proteins) absorbed radiation in multiple regions including two strong bands at 1500−1690 cm^−1^ (Figure 4B) [22]. The Raman spectroscopy results evinced homogeneous chemical composition of the PLCL/PCL nanofibrous membrane. The analysis of a single PLCL/PCL fibre indicated the presence of both polymers (Supplementary Material Figure S3).

The nanofibrous materials analysed were porous and hydrophilic, and therefore it was not possible to measure equilibrium contact angles of the droplets deposited on the surface of these materials [23,24]. However, the wettability of the pure NF and lyophilized coated NF was characterized by measuring time between the deposition and complete penetration of a water droplet into the membranes. Results showed slow penetration of water droplet into pure NF (10.9 ± 0.3 s) compared to NF0 (4.4 ± 0.3 s) and NF1 (2.9 ± 0.5 s).

### 3.3. Monoculture of HaCaT Keratinocytes on the NF

**Figure 5:**
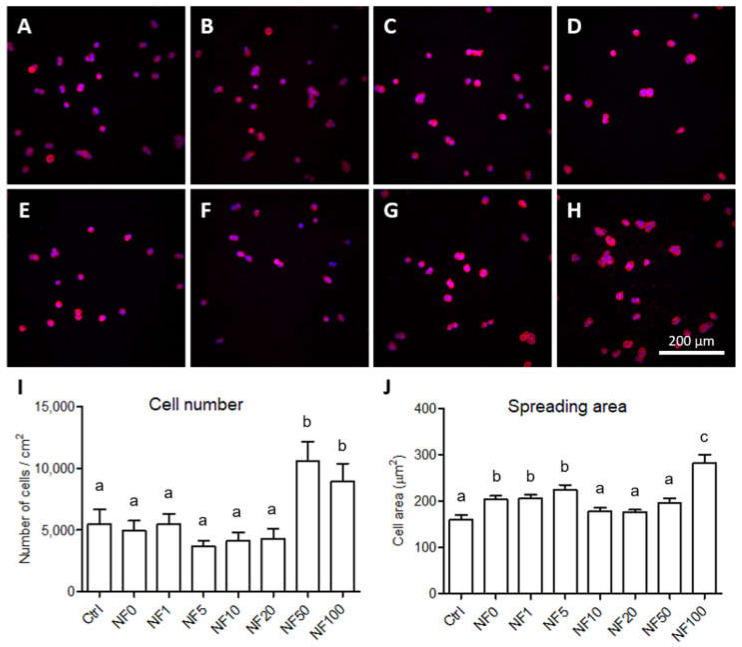
HaCaT cells that adhered to the pure NF membranes (Ctrl, **A**) and to the NF membranes with fibrin assemblies containing 0–100 vol.% of PL, i.e., NF0 (**B**), NF1 (**C**), NF5 (**D**), NF10 (**E**), NF20 (**F**), NF50 (**G**) and NF100 (**H**) on day 1 following seeding. Images of the cells stained with phalloidin-TRITC (red); the cell nuclei were counterstained with Hoechst 33,258 (blue). Leica SPE microscope, obj. x 20. The cell population density (**I**) and the spreading area (**J**) of the HaCaT cells on the NF membranes on day 1 following seeding. The data are expressed as the mean ± standard error of the mean. The superscript letters above the columns denote significant differences between the NF membranes that do not share the same superscript (*p* < 0.05).

The adhesion and cytoskeleton organization of the cells that grew on the samples were evaluated after 24 h in the culture via the staining of the stress fibres and the cell nuclei. The results indicated that the keratinocytes were able to adhere to all the samples and evinced a rounded shape, which is the normal morphology of keratinocytes after 24 h of culturing. In addition, it was possible to detect small clusters of cells in certain regions that had created islands, behaviour that is typical of keratinocytes (Figure 5A–H). The quantification of the number of cells that grew on the samples revealed that a significantly higher number of HaCaT cells had adhered to the NF50 and NF100 membranes (Figure 5I). The spreading area of the cells that adhered to the NF membranes was also quantified after 24 h of culturing and, similarly, the HaCaT cells evinced a significant increase in the spreading area when cultured on the NF100 membranes. In addition, the spreading area of the cells grown on the NF0, NF1 and NF5 membranes was greater than that on the pure nanofibres (control), but significantly lower than on the NF100 membranes (Figure 5J).

**Figure 6:**
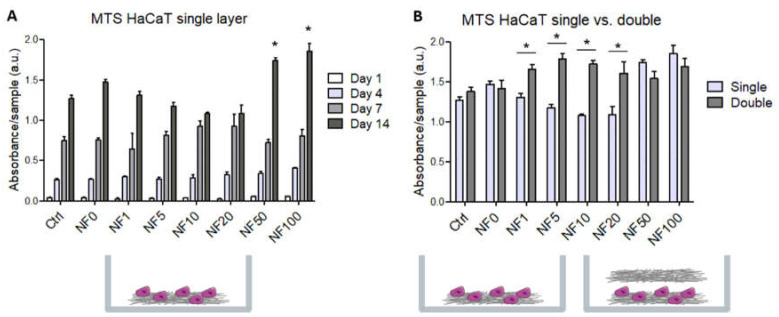
Metabolic activity/viability of the HaCaT cells that grew on the NF membranes with fibrin assemblies prepared using differing platelet lysate (PL) concentrations, i.e., 0, 1, 5, 10, 20, 50 and 100% PL (NF0, NF1, NF5, NF10, NF20, NF50, NF100, respectively). Pure NF membranes were used as the control sample (Ctrl). (**A**) HaCaT cells were grown on a single layer of NF for 1, 4, 7 and 14 days. The asterisks above the columns indicate significant differences compared to the control membrane (*p* < 0.05). (**B**) Comparison of the cells that grew on the NF membranes for 14 days (single) and the cells that grew on the NF membranes in a medium to which additional NF membranes with corresponding PL concentrations were added on day 7 following seeding (double). The asterisks above the columns indicate significant differences between the single and double layers (*p* < 0.05).

In addition, the metabolic activity of the HaCaT cells cultured on the NF membranes for 1, 4, 7 and 14 days was quantified. The cells were found to be able to grow and proliferate on all the tested NF membranes without any signs of cytotoxic or cytostatic effects. The enhanced metabolic activity of the keratinocytes was observed on the NF50 and NF100 membranes compared to that of the control membranes on day 14 (Figure 6A).

An additional NF layer was emplaced in the cell culture on day 7 following seeding in order to enhance the effect of the PL on the HaCaT cells, and the cell viability/metabolic activity was evaluated on day 14 following seeding and compared with the single layer results. Figure 6B illustrates that the additional layer enhanced the metabolic activity in those samples that contained low percentages of PL, i.e., NF1, NF5, NF10 and NF20. However, with respect to NF50 and NF100, which contained the highest amounts of PL, as well as both of the control samples without PL, i.e., the Ctrl and NF0 samples, the additional layer did not act to further enhance the metabolic activity of the HaCaT cells compared to the activity observed with the single layer.

**Figure 7:**
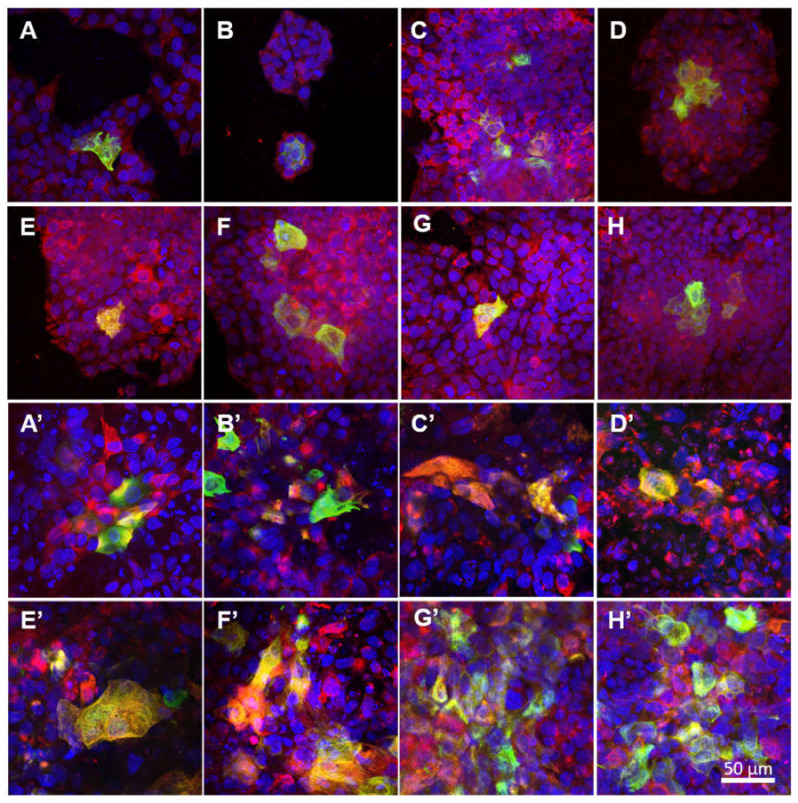
Immunofluorescence staining of the cytokeratin 10 (green) and cytokeratin 14 (red) in the HaCaT cells in the monoculture for the pure nanofibres (**A**,**A’**) and nanofibrous membranes with differing PL concentrations, i.e., NF0 (**B**,**B’**), NF1 (**C**,**C’**), NF5 (**D**,**D’**), NF10 (**E**,**E’**), NF20 (**F**,**F’**), NF50 (**G**,**G’**), NF100 (**H**,**H’**) (**A**–**H** on day 7 and **A’**–**H’** on day 14). The cell nuclei were counterstained with Hoechst (blue). Leica SP8 confocal microscope, obj. × 20.

The differentiation of the keratinocytes that grew in the monoculture on various NF membranes was analysed via the immunofluorescence detection of cytokeratin 14 and cytokeratin 10 on day 7 (Figure 7A–H) and day 14 (Figure 7A’–H’) after seeding. Different types of cytokeratin were found to be present according to the differentiation stage of the cells. It is known that in the physiological epidermis, cytokeratin 14, which is associated with cell proliferation, is present in the basal layer of keratinocytes, whereas cytokeratin 10 is present in the upper layers of cells in the early differentiation stage.

The positions of the cells in the samples that presented the two types of cytokeratin on the *z*-axis was determined via the confocal microscopy technique, which enabled the determination of a series of horizontal optical sections through the samples, followed by the 3D reconstruction of the cell-NF membrane complex. It was observed that the first layer of HaCaT cells that grew directly on the NF membranes was positively stained for basal cytokeratin 14, while the more differentiated HaCaT cells that were positively stained for cytokeratin 10 appeared in the second layer of the HaCaT cells that grew on the basal layer of the cells (Figure 7). From day 7 to day 14, the staining for cytokeratin 10 became brighter and more pronounced, especially concerning the cells on the NF membranes with higher concentrations of PL (20–100%). On day 14 following seeding, the highest amounts of cells that were positive for cytokeratin 10 was determined on the NF50 and NF100 samples (Figure 7G’,H’), which correlated to the highest metabolic activities of the cells on these membranes.

### 3.4. Metabolic Activity of the Keratinocytes Co-Cultured with Endothelial Cells

Two different cell types that participate in the wound healing process, HaCaT keratinocytes and endothelial HSVEC cells, were co-cultured aimed at analysing the paracrine effect of the endothelial cells on the response of the HaCaT keratinocytes to the tested NF membranes. The HaCaT cells were seeded directly on the membranes, which were then placed in inserts, whereas the HSVEC were cultured on the bottoms of the wells of a cell culture plate without physical contact with the membranes. The metabolic activity of the HaCaT and HSVEC cells in both the monocultures and co-cultures was analysed on days 7 and 14 following seeding.

**Figure 8:**
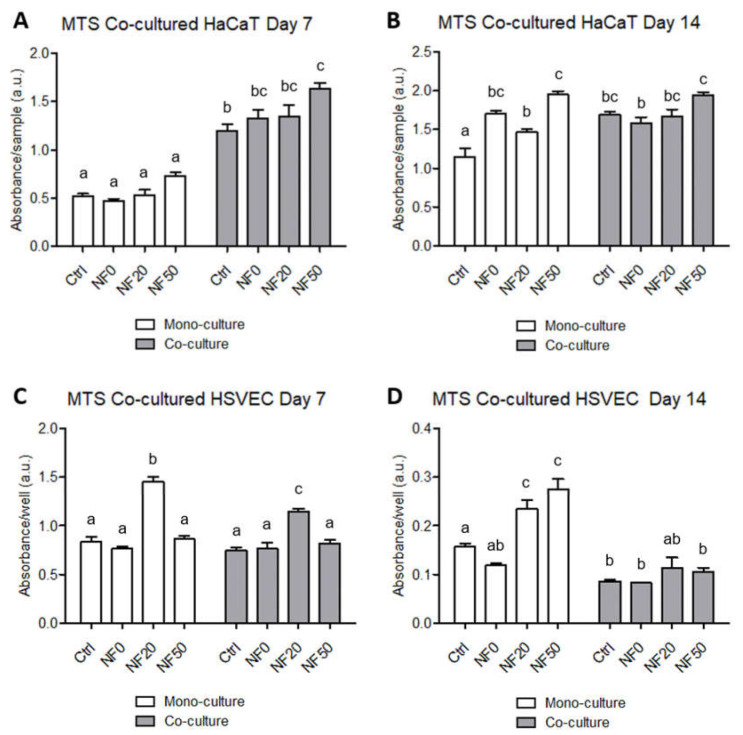
The metabolic activity of the HaCaT and HSVEC in the monocultures and co-cultures of these cell types. The metabolic activity of the HaCaT cells (**A**,**B**) that grew on the pure NF membranes (Ctrl), and on the NF membranes with fibrin assemblies prepared using various platelet lysate (PL) concentrations, i.e., 0%, 20% and 50% PL (NF0, NF20 and NF50, respectively). The metabolic activity of the human saphenous vein endothelial cells (HSVEC) (**C**,**D**) that grew on the well bottoms on a polystyrene well plate in the presence of the Ctrl, NF0, NF10, NF20 and NF50 membranes after 7 days (**A**,**C**) and 14 days (**B**,**D**) of cultivation. Each graph contains information on the cells that grew in the monoculture (white columns) and those co-cultured with the other cell type (grey columns). The superscript letters above the columns denote significant differences between the NF membranes that do not share the same superscript (*p* < 0.05).

The experimental results revealed the positive effect of HSVEC on the metabolic activity of the HaCaT for all the samples on day 7 following seeding (Figure 8). As anticipated, no significant differences in the HaCaT cell metabolic activity were observed between the various NF samples after 7 days in the monoculture (cf. Figure 6A and Figure 8A). However, with respect to the co-culture with HSVEC, a significant increase in the metabolic activity of the HaCaT cells was observed on the NF50 membrane compared to all the other NF membranes on day 7 (Figure 8A). On the other hand, after 14 days in the culture, the positive effect of HSVEC on the metabolic activity of the HaCaT was observed only for the pure nanofibres (Ctrl), i.e., not for any of the NF membranes modified with fibrin or with fibrin + PL. As on day 7, the highest HaCaT cell absorbance values were observed on the NF50 membranes (Figure 8B).

Interestingly, the HSVEC metabolic activity results indicated that the co-cultivation of these cells with HaCaT did not lead to an increase in their proliferation after 7 days in the culture. The highest values were observed for the HSVEC cells cultured with the NF20 membranes (Figure 8C). However, the data from the 14th day of the co-culturing of HSVEC with the HaCaT revealed an obvious decrease in the metabolic activity of the endothelial cells compared to both results obtained on day 7 and the HSVEC values in the monocultures. The metabolic activity in the 14-day-old HSVEC monocultures increased slightly following the culturing of the cells with the NF20 and NF50 membranes when compared with the control membranes (Figure 8D).

### 3.5. Differentiation of the Keratinocytes Co-Cultured with Endothelial Cells

**Figure 9:**
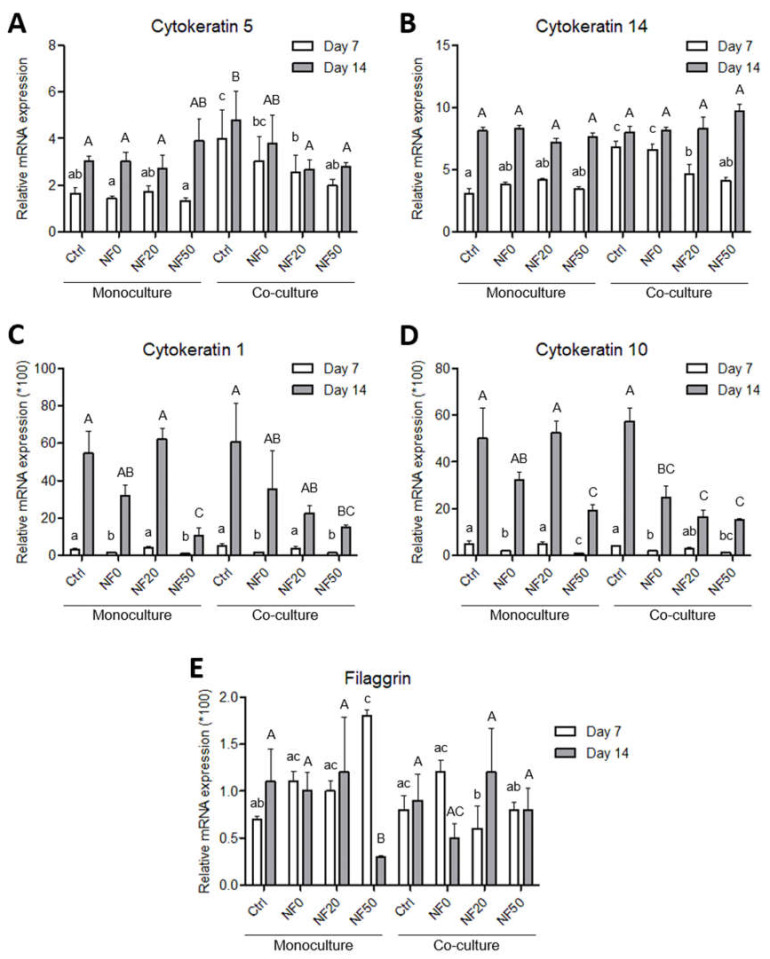
Quantification of the mRNA levels of the HaCaT in the monoculture and the co-culture with HSVEC. The relative expression of cytokeratin 5 (**A**), cytokeratin 14 (**B**), cytokeratin 1 (**C**), cytokeratin 10 (**D**) and filaggrin (**E**) on days 7 and 14 following seeding on the NF membranes. The target gene levels are expressed as relative values towards the reference B2M gene. The differing superscripts above the columns denote significant differences between the membranes that do not share the same superscript (*p* < 0.05) for each cell culture type separately (monoculture and co-culture). The lower case letters denote significant differences on day 7, the upper case letters on day 14.

The influence of the tested NF membranes on the gene expression in the cells was evaluated using both the monoculture and co-culture systems. Cytokeratin 5 and cytokeratin 14 comprise specific intermediate filament components in the keratinocytes of the basal layer. After 7 days in the culture, the expression levels of mRNA for cytokeratin 5 and cytokeratin 14 were observed to be significantly higher in the HaCaT cells co-cultured with HSVEC than in the mono-cultured cells from the NF membranes without PL, i.e., the pure membranes (Ctrl) and NF0 (Figure 9A,B). In the case of cytokeratin 5, this trend persisted up to day 14 of cultivation, although no differences were observed when the pure membranes in the co-culture were compared to the NF50 in the monoculture (Figure 9A). However, in the case of cytokeratin 14, similar expression levels were attained following 14 days of culturing with respect to all the tested NF membranes (Figure 9B).

Cytokeratins 1 and 10 were analysed as early keratinocyte differentiation markers. As is evident from Figure 9C,D, both cytokeratins evinced similar expression patterns in response to the variations in the NF membrane composition and the culture conditions, i.e., the monoculture versus the co-culture. After 7 days of cultivation, the expression levels of both cytokeratins were relatively low and did not evince any significant differences when the monocultured and co-cultured cells were compared (Figure 9C,D). With respect to the NF membrane composition, the expression of cytokeratins 1 and 10 was significantly higher on the cells grown on the NF20 samples than on the NF0 and NF50 samples following 7 days of culturing. After 14 days, a noticeable increase was observed in the expression of both cytokeratins on all the samples. The highest expression values in the monocultures were again evinced by the NF20 samples. However, the highest expression values in the co-cultures were observed for the cells cultured on the pure nanofibres, with a tendency for the expression to decrease with increasing PL concentrations (Figure 9C,D).

The assessment of the expressions of filaggrin and involucrin as late keratinocyte differentiation markers revealed relatively low expression levels in the HaCaT cells (Figure 9E). On day 7, the filaggrin expression attained its highest level in the cells on the NF50 membranes in the monocultures, where it was found to be significantly higher than the values for the pure membrane in the monoculture and the NF20 and NF50 in the co-culture. However, after 14 days, the expression of filaggrin in the monocultured cells on the NF50 decreased markedly so as to attain one of the lowest values. With concern to the co-cultures, the expression of filaggrin was generally similar or lower than in the monocultured cells with no clear trend being evident. From day 7 to 14, the expression decreased in the cells on the NF0, while it evinced an increasing tendency in the cells on the NF20 and remained the same on the control pure NF and NF50. The expression of involucrin was very low and was found to be similar in the HaCaT cells of all the tested samples in both culture systems and at both time intervals (data not shown).

**Figure 10:**
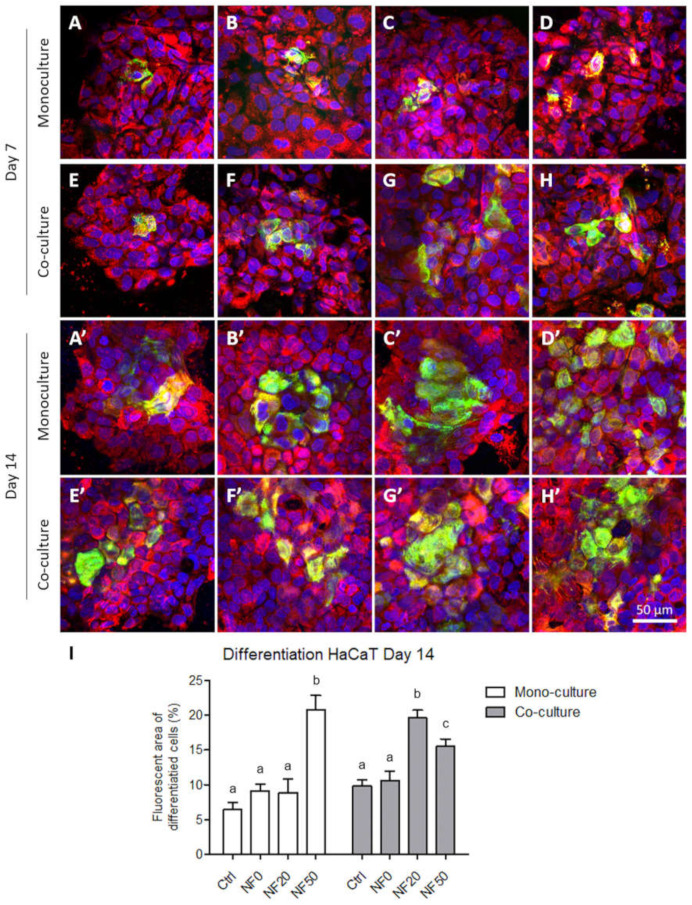
Differentiation of the HaCaT cells in the monoculture and co-culture on day 7 (**A**–**H**) and on day 14 (**A’**–**H’**). The HaCaT cells monocultured on the NF samples (**A**–**D**,**A’**–**D’**) and co-cultured with HSVECs (**E**–**H**,**E’**–**H’**) in the presence of the membranes as follows: Ctrl (**A**,**E**,**A’**,**E’**), NF0 (**B**,**F**,**B’**,**F’**), NF20 (**C**,**G**,**C’**,**G’**) and NF50 (**D**,**H**,**D’**,**H’**). The cells were stained with immunofluorescence for cytokeratin 10 (green) and for cytokeratin 14 (red). The cell nuclei were counterstained with Hoechst 33,258 (blue). Leica SP8 confocal microscope, obj x 20. Area (%) of the image covered with cells positive for cytokeratin 10 on the pure membranes (Ctrl), on the membranes with fibrin (NF0) and on the membranes with fibrin and 20% PL or 50% PL (NF20 and NF50) samples, analysed in the monoculture (white columns) and co-culture (grey columns) (**I**). The differing superscripts above the columns denote significant differences between the membranes that do not share the same superscript (*p* < 0.05).

The presence of cytokeratin 14 and 10 in the keratinocytes at the protein level was analysed by means of immunostaining in the monocultures and co-cultures of the HaCaT keratinocytes with endothelial HSVEC cells on days 7 and 14. On day 7, keratinocytes positive for cytokeratin 10 were observed on all the tested membranes in the form of islands on the monolayers of the keratinocytes positive for cytokeratin 14 (Figure 10A–H). From day 7 to day 14, the area covered by the keratinocytes positive for cytokeratin 10 generally increased, a factor that was more pronounced for the keratinocytes co-cultured with the endothelial cells. With respect to the monoculture group, this area expanded significantly more on day 14 in the NF50 samples than in the other samples of the same group, while concerning the co-culture group, this area was observed to be significantly larger in the NF20 and NF50 samples than in the other samples from this group (Figure 10A’–H’,I).

### 3.6. Endothelial Cell Maturation and Migration towards the NF Membranes That Contained Platelet Lysate

**Figure 11:**
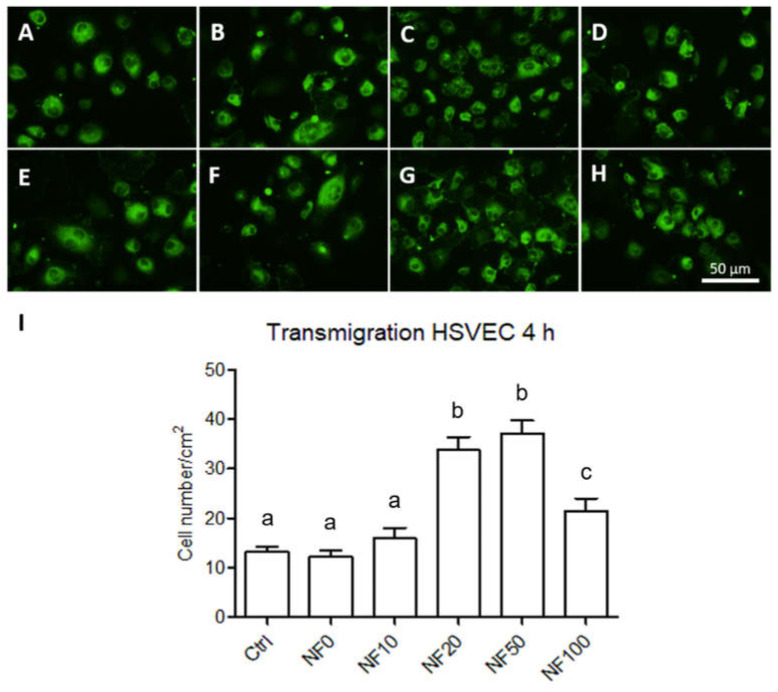
The maturation of the HSVECs in the monoculture and co-culture on day 7. The HSVECs monocultured in polystyrene wells with NF membranes floating in the medium (**A**–**D**) and co-cultured with HaCaT (**E**–**H**) grown on the membranes. In both cases, the following membranes were used: Ctrl (**A**,**E**), NF0 (**B**,**F**), NF20 (**C**,**G**) and NF50 (**D**,**H**). The cells were stained with immunofluorescence for the von Willebrand factor (green); Olympus IX71 microscope (Olympus, Tokyo, Japan), DP80 digital camera (Olympus, Tokyo, Japan), obj. × 20. The number of HSVECs that transmigrated through the inserts towards the medium with the Ctrl, NF10, NF20, NF50 and NF100 samples over 4 h (**I**). The differing superscripts above the columns denote significant differences between the samples that do not share the same superscript (*p* < 0.05).

The differentiation/maturation status of the HSVECs was analysed via the immunofluorescence staining of the von Willebrand factor in the HSVEC monoculture and in the co-culture with HaCaT keratinocytes according to the scheme in Figure 2 on day 7 following seeding. The staining pattern of the HSVECs appeared to be similar for all the assessed membranes, with no clear differences between them for either the monocultures (Figure 11A–D) or co-cultures (Figure 11E–H).

The migration of the HSVECs towards the wound site comprises an important event in the wound healing process in terms of initiating vascularization. The HSVEC migration towards the different NF membranes was quantified after 4 h in the culture. As can be observed in Figure 11I, the number of HSVECs cells that crossed the insert membrane was significantly higher in the wells with the addition of NF 20, NF50 and NF100 samples than in those with the control and NF0 samples. The highest numbers of transmigrated cells were observed following the addition of NF50 and NF 20 membranes to the medium.

## 4. Discussion

Reduced vascular flow acts to prevent wound healing in diabetic patients and other patients with vascular diseases. The new PLCL/PCL nanofibrous membranes (NF) with fibrin assemblies containing platelet lysate (PL) should mechanically prevent microbial infections, drain the exudate and contribute to skin regeneration via the release of bioactive compounds from the PL. The present work was mainly focused on the membranes’ effect on keratinocytes and endothelial cells, which are involved in the skin wound healing process. We marginally mentioned the increase capillary penetration of water into coated NF, which may be beneficial for drain the exudate. The coating formation mainly at the surfaces of individual fibres makes it possible to apply the coating technology to an optimum PLCL/PCL without changing its porosity. In addition, the coating was stable for at least 7 days in PBS.

The PLCL/PCL nanofibres were prepared using the needleless electrospinning technique. A polymer solution was applied to the wire electrode and a nanofibre layer was produced due to electrostatic forces. The parameters were optimized with respect to the optimal spinnability (i.e., the fibre diameter, minimum distribution curve, absence of bead defects, etc.). In addition, the distance between the wire and the collector was chosen according to the final fibre morphology, avoiding bead defects and the evaporation of the solvent before the fibres were deposited on the collector. It is important to take into account that too short a distance between the wire and the collector induces the formation of fibres coated with solvent residues and wet drops on the collector. Conversely, too great a distance results in the fibres not being able to reach the collector. Moreover, the solvent used has the potential to influence the morphology of the electrospun fibres. The solvent system used for the PLCL/PCL electrospinning contained chloroform, ethanol and acetic acid and was studied previously by our group [18,19,25,26]. The addition of acetic acid to the normally applied system (chloroform:ethanol) served to enhance the fibre diameter and the fibre diameter distribution width and led to the avoidance of the formation of bead defects.

The fibre diameter range obtained for the PLCL/PCL-blend nanofibres was compared to pure PCL and pure PLCL nanofibres (Supplementary Material Figure S4). The pure PLCL and PLCL/PCL blend showed similar fibre diameter distribution, although pure PCL showed a smaller distribution range. We hypothesize that weaker interactions between PLCL random co-polymer molecules is mainly the cause of the wider diameter distribution range.

The chemical composition of nanofibrous membranes was measured on different lots of materials (Supplementary Material Figure S5). The results obtained clearly demonstrate the reproducibility of the method used. The FTIR spectra differ only minimally, and the content of both polymers within the separately prepared mats was similar. Moreover, Raman spectroscopy confirmed the presence of PLCL and PCL in a single fibre. These results show the homogenous distribution of the components.

PL contains a mixture of various growth factors, cytokines and other bioactive molecules and ions, which play an important role in the wound healing process [27]. We analysed the concentration of three growth factors present in the PL and determined values for PDGF (~16.4 ng/mL), FGF (~62.9 pg/mL) and VEGF (~51.2 pg/mL). Zamani et al. (2019) compiled data from several studies that determined ranges of 3.3–24 ng/mL for PDGF, 0.07–0.57 ng/mL for FGF and 0.1–11 ng/mL for VEGF [28]. Whereas our results were in accordance with the ranges reported in the literature for PDGF, reduced values were observed for FGF and, especially, for VEGF. Our study included the preparation of NF containing differing concentrations of proteins from fibrinogen solutions loaded with various concentrations of PL. Even though FGF and VEGF are capable of accumulation in fibrin via specific binding centres in the fibrin [29], they were not detected in the NF20, most probably due to the relatively low concentrations of these growth factors in the PL (FGF, ~63 pg/mL and VEGF, ~51 pg/mL). The PDGF-BB concentration of 16.7 pg/cm^2^ detected in the NF20 most likely originated from the elevated PDGF-BB concentration, i.e., 16 ng/mL in the PL. Since the total protein content increased proportionally to the amount of PL added during the fibrin assembly preparation process, the samples with higher concentrations of PL, i.e., the NF50 and NF100, could have been expected to contain a higher amount of each of the growth factor/bioactive compounds. The PDGF-BB at least was capable of participating in the significant beneficial effect of the PL in the NF50 and NF100 on the seeded HaCaT on these samples [30], together with other growth factors such as epidermal growth factor, which is usually present in PL [27]. The positive effect of the PL on the metabolic activity/viability of the HaCaT cells is illustrated in Figure 6. Bioactive compounds may have been released from the PL via its dissolution with the cultivation medium [31] and/or via the degradation of the fibrin coating by plasmin. Plasmin, i.e., a serine protease which exhibits fibrinolytic activity, may have been produced via the activation of plasminogen that originated from the immobilized PL and the serum in the cultivation medium following its binding to specific receptors on the HaCaT cell surface [32].

The first stage of the in vitro experiments with the NF membranes comprised the analysis of the effect of various concentrations of the PL on keratinocytes. Anchorage-dependent cells, including keratinocyte cells, must adhere in order to resume the cell cycle and proliferate. PCL is known to be more hydrophobic (an unfavourable factor in terms of support for cell adhesion) than other commonly used synthetic degradable polymers (e.g., PLA, PLGA) [33,34]. This disadvantage with regard to PCL can, however, be mitigated via the co-polymerization of ɛ-caprolactone with L-lactic acid, following which the resulting PLCL copolymer presents more favourable surface properties in terms of cell adhesion due to its enhanced hydrophilicity [4,35]. The results concerning the initial adhesion and morphology of the HaCaT cells indicated that all the tested NF membranes produced via the combination of PLCL and PCL allowed for an adequate degree of cell attachment and spreading. The cell behaviour was further improved via the modification of the NF membranes with PL. This was documented by the higher number of cells that attached to the NF50 and NF100 membranes than to the membranes without PL, as well as by the enhanced spreading of the cells on the NF100 during the first 24 h of experimentation. The study employed a wide range of PL concentrations (1 to 100 vol.%) in the preparation of the NF membranes. Moreover, selected cell cultures were supplied with additional membranes after 7 days of cell cultivation, which allowed for the observation of the prolonged effect of the PL components released on the proliferation and differentiation of the keratinocytes. The quantification of the metabolic activity over time demonstrated the proliferation of the cells up to 14 days in the culture on the membranes. The absence of cytotoxic and cytostatic effects was evidenced by the fact that the HaCaT cells were able to proliferate on all the samples. These results are in agreement with previous findings that demonstrated the safety of PLCL and PCL polymers [35,36,37]. Similarly, the fibrin assemblies were found to be cytocompatible with various other cells types, e.g., endothelial cells [38,39] and fibroblasts [40].

In addition, the metabolic activity results revealed that the NF50 and NF100 membranes served to enhance cell proliferation. Several authors that have added PL directly to culture media [5,14,15,41] have observed the positive effect of PL on cell proliferation. With respect to our study, PL in various concentrations was assembled with fibrin. The positive effect on cell proliferation was observed due to the release of the growth factors present in the PL onto the nanofibrous membranes, and it was noted that even a small amount of PL attached to the NF membranes led to the enhancement of the proliferation of keratinocyte. The addition of extra NF membranes on day 7 resulted in an increase in the metabolic activity of the HaCaT cells on all the samples that contained PL, with the exception of the NF50 and NF100 samples, i.e., the samples with the highest PL concentrations, which most likely ensured the maximum stimulation of cell proliferation unaided. The results demonstrate the clear positive effect of membranes containing PL on keratinocyte proliferation, which is important in terms of skin wound regeneration. In addition, we assume, based on our experiments with additional membranes, that the maximum stimulatory effect of PL was attained with respect to the NF50 and NF100 samples.

The epidermis comprises a stratified tissue composed of keratinocytes in several differentiation stages. In this respect, cytokeratins, a distinct component of the intermediate filaments, exhibit a complex expression pattern that is regulated by the stage of tissue differentiation. Specifically, cytokeratins 5 and 14 are present in the basal proliferating layer, whereas cytokeratins 1 and 10 are present in the upper (suprabasal) layers of early differentiation. In addition, filaggrin and loricrin are considered late differentiation keratinocyte markers [42]. Our results on the immunodetection of cytokeratins 10 and 14 indicated that the HaCaT cells were able to differentiate on the NF membranes prepared in this study. In addition to its positive effect on the cell metabolic activity, PL also exerted a stimulatory effect on keratinocyte differentiation. Both the NF50 and NF100 membranes served to increase the number of differentiated cells positively stained for cytokeratin 10 (as estimated by the area covered with cytokeratin 10-positive cells) as compared to the membranes with lower PL contents.

Keratinocytes and endothelial cells comprise the two dominant cell types in skin tissue, and both of them contribute to the wound healing process. The co-culturing of the two cell types in vitro allowed for the analysis of the paracrine effect of both cell types, as well as for the direct interaction of the HaCaT cells with the NF membranes. For the purposes of this study, the co-cultures were developed using inserts, thus allowing for spatial compartmentalization. The two cell types shared the same culture medium, but were not in direct physical contact in order to avoid potential mismatches in the subsequent analysis of the cell metabolic activities and cell differentiation markers. The keratinocytes were cultured directly on the membranes, whereas the endothelial cells were cultured in indirect, i.e., remote and exclusively humoral, contact. The positive effect of the NF membranes containing the PL on the proliferation of the HaCaT cells was further enhanced by the presence of HSVEC in the co-culture. Conversely, however, the proliferation of HSVEC was limited by the presence of the HaCaT, and this effect was observed to increase with time. In order to better elucidate the role of the PL and the endothelial cells, each membrane containing differing percentages of PL was analysed in a keratinocyte monoculture and in a co-culture system with endothelial cells. In both cases, the only difference in the setup concerned the absence or presence of endothelial cells. On day 7, a reduction in the metabolic activity of the HSVEC in the co-culture compared to the corresponding cells in the monoculture was observed only with respect to the NF20 sample; however, after 14 days, a decrease in the metabolic activity was observed in the cultures with three of the samples, i.e., the control, NF20 and NF50. Endothelial cells require a cell culture medium supplemented with several growth factors, such as the commercially available EGM-2 medium, in order to ensure the appropriate growth. Our previous experiments on HSVEC proliferation employing a weak medium indicated a reduction in the cell proliferation after 7 days of culturing due to the limitation of nutrients in the medium. However, by using a medium without growth factors, we attempted to mimic a chronic wound environment in which the presence of growth factors is limited [10]. The data presented suggest that the availability of nutrients is reduced when HSVECs are co-cultured with HaCaT cells due to the high consumption of such nutrients by the HaCaT that, in addition, exhibited higher metabolic activity in the co-cultures than in the monocultures. Nevertheless, the metabolic activity of the keratinocytes was enhanced by the presence of endothelial cells, with respect to which these cells exhibited their maximum metabolic activity.

It has previously been described that co-cultures of various cell types influence the responses of the individual cell types to various stimuli. Several authors have analysed in vitro interactions between keratinocytes and fibroblasts in correlation with skin wound healing [43,44]. However, to the best of our knowledge, very few studies have analysed the interaction of keratinocytes and endothelial cells in a co-culture in vitro. Although Baltazan et al. (2019) developed a multi-layer skin structure using keratinocytes and endothelial cells, among other cell types, the behaviour of the individual cell types when co-cultured or monocultured was not the subject of the manuscript [45]. According to our results, we hypothesize that the extracellular matrix, cytokines and growth factors produced by endothelial cells are responsible for the enhanced metabolic activity observed on the keratinocytes. Endothelial cells produce fibroblast growth factors, particularly FGF-2, which serve to enhance the cell motility and re-epithelialization process via cell proliferation. In addition, endothelial cells produce granulocyte macrophage-colony stimulating factor and stromal cell-derived factor 1, which mediate the proliferation of keratinocytes [10,46]. Conversely, keratinocytes produce several growth factors and cytokines which are also synthesized by endothelial cells, such as PDGF, VEGF and FGF [46,47]. The presence of growth factors in a co-culture medium conditioned by endothelial cells has the potential to up-regulate the synthesis of bioactive molecules in keratinocytes and thus increase their metabolic activity.

The development of an in vitro system for the co-cultivation of two or more differing types of cell is a complicated task. Each cell type has its own culture medium requirements (e.g., different amounts of serum and growth factors). Our study involved the development of a system that applied a mixture of keratinocyte and endothelial cell culture media which enabled the proliferation of both cell types. It is important to note, however, that the endothelial cells were cultured in a relatively weak medium with reduced amounts of the recommended supplements or even with no supplements at all. Nevertheless, the weak culture medium employed allowed for the analysis of the proliferation and differentiation of keratinocytes without the disruption of these cellular processes by various less-defined factors. The results of the experiments in which HaCaT cells were cultured either in a keratinocyte medium or in a mixture of endothelial and keratinocyte media did not reveal any considerable differences in terms either of the cell response or the effect of NF membranes on the cell proliferation and differentiation.

The differentiation of the keratinocytes was analysed via the expression of the genes for the proteins involved in stratification and via the detection of two of these proteins by means of immunostaining. The expression levels of five proteins were analysed following monoculturing and the co-culturing of the keratinocytes with endothelial cells. We quantified the expression of two basal cytokeratins (5 and 14), two early differentiation cytokeratins (1 and 10) and filaggrin as a late differentiation marker. The expression pattern was, in most cases, found to be similar for the keratin pairs, and was related to the intracellular pathways involved in either proliferation or differentiation. In this respect, the expression of the basal cytokeratins served to block the expression of cytokeratin differentiation, whereas a switch to differentiation pathways brought about a reduction in the expression of the basal cytokeratins [48].

The results concerning basal cytokeratins 5 and 14 indicated the positive effect of the endothelial cells in the co-culture on the expression of specific intermediate filaments so as to form a basal layer of keratinocytes during the first days of culture. These results were in accordance with the increased metabolic activity observed after 7 days in the co-cultured system. However, the expression of the early differentiation markers (cytokeratins 1 and 10) at the mRNA level was not enhanced by the presence of the endothelial cells in the co-culture although, as mentioned above, the area of the membranes covered with cytokeratin 10-positive cells was higher on the NF20 in the co-culture than in the monoculture on day 14. This disproportion may have been due to the fact that the expression at the mRNA level reflects the current gene expression status in the cell, whereas the total amount of the protein in the cell is usually the result of long-term accumulation. The expression of mRNA precedes proteosynthesis, and an increased amount of a synthesized protein may exert a negative effect on the mRNA expression. A similar disproportion in the expression of talin and vinculin, i.e., proteins that participate in cell adhesion, at the mRNA and protein levels was observed in a recent study performed on human osteoblast-like cells in cultures on nanofibrous PLLA scaffolds reinforced with diamond nanoparticles [49].

With regard to the immunostaining of cytokeratin 10, the co-cultured keratinocytes that grew on the NF20 and NF50 evinced enhanced differentiation values compared to the control pure membranes, whereas the mono-cultured keratinocytes evinced enhanced values on the NF50 sample only. Taking the metabolic activity of the endothelial cells together with the keratinocyte differentiation, it is reasonable to assume that the enhanced metabolic activity of the endothelial cells in the presence of the NF20 membranes exerted a positive effect on keratinocyte differentiation. It can be expected that this paracrine effect, together with the effect of the PL-loaded membranes, will act synergistically to enhance the wound healing process. Filaggrin, a late marker of differentiation, evinced high expression levels in the NF50 monocultures after 7 days of culturing, although the expression thereof declined after 14 days of culturing. These results indicate that the membranes containing PL acted to enhance differentiation during the first few days of culturing.

The detection of the von Willebrand factor indicated that all the tested membranes allowed for the phenotypic maturation of the endothelial cells. The von Willebrand factor is a specific marker of endothelial cells, which plays an important role in the adhesion of platelets to the vascular wall and the wound site [50]. The primary human endothelial cells used in our experiments evinced a basal amount of von Willebrand factor without the need for a complementary differentiation medium. Thus, the presence of the von Willebrand factor after 7 days of culturing indicated that the co-culture containing keratinocytes and NF membranes did not interfere with the endothelial cell maturation process. These results are in agreement with those of other authors who indicated that PL acts to enhance endothelial cell proliferation without affecting their differentiation capacity [51].

Finally, angiogenesis is crucial to the wound healing process as a result of the formation of new capillaries. It is known that a reduction occurs in angiogenesis in chronic wounds [52]. In this respect, the migration of endothelial cells to the wound site is essential so as to ensure the wound healing process. We therefore assessed the potential of our PL-containing membranes to enhance endothelial cell migration. The results revealed that the NF20, NF50 and NF100 membranes enhanced the migration of HSVEC towards the media with the membranes; the maximum number of migrated cells were observed in the wells with the NF20 and NF50 samples. These results demonstrate the chemotactic effect of the PL released from the membranes on the endothelial cells. The effects of PL in various types of angiogenesis assays have previously been analysed by several authors [41,51]. The effects include the promotion of the effect of PL on the migration and tubule-forming capacity of endothelial cells. In our case, while the PL was not freely available in the cell culture medium, it was assembled with fibrin on the NF membranes. Thus, the NF membranes containing at least 20% of PL when fabricated were able to release part of the PL and to attract endothelial cells to the wound dressing site.

## 5. Conclusions

The study involved the preparation of nanofibrous PLCL/PCL membranes by means of electrospinning, which were then coated with fibrin assemblies loaded with various concentrations of PL in order to obtain a novel potential candidate for skin wound healing dressings.

The pure nanofibrous membranes with and without fibrin assemblies and the nanofibrous membranes containing PL allowed for the adhesion, proliferation and differentiation of human HaCaT keratinocytes without exerting any cytotoxic effects over time. Increases in the PL content in the fibrin assemblies acted to enhance the proliferation and differentiation of keratinocytes, with the maximum effect being observed at 50–100% of PL. The membranes containing a medium PL content, i.e., 20–50% of PL, resulted in the most effective enhancement of the proliferation and migration of endothelial cells.

The keratinocyte and endothelial cell co-culture system allowed for the clarification of the interactions between the two components. The presence of endothelial cells in the cell culture enhanced the proliferation and differentiation of keratinocytes during the first week of culturing, with the maximum effect being observed with respect to the membranes with 20–50% of PL.

Thus, the beneficial effect of biodegradable polymeric membranes with fibrin assemblies and PL was demonstrated employing keratinocytes, endothelial cells and co-cultures thereof in vitro. Hence, we propose that these materials, especially NF20 and NF50, have the potential to contribute towards the effective treatment of chronic wounds.

## Data Availability

Data is contained within the present article or Supplementary Material.

## Supplementary Materials

The following are available online at https://www.mdpi.com/2079-4991/11/2/457/s1, Figure S1: SEM images of the nanofibrous membranes: PLCL/PCL membrane (A), lyophilised PLCL/PCL membrane coated with fibrin (B) and lyophilised PLCL/PCL membrane coated with fibrin containing 50% of platelet lysate (C), Figure S2: CLSM images of the fluorescence-stained proteins on the upper surface of the NF modified from a solution containing Fbg and 50% PL (NF50) observed in PBS after the modification (A) and after one week in PBS (B), Figure S3: Raman spectroscopy analysis: (A) picture of analysed area, (B) obtained Raman shifts of pure PCL, pure PLCL and their PCL/PLCL blend: 3387 cm^−1^—Stretching vibr. of C-H (PLCL), 2944 cm^−1^—Stretching vibr. of C-H (PLCL), 2915 cm^−1^—Stretching vibr. of C-H (PLCL, PCL), 2873 cm^−1^—Stretching vibr. of C-H (PLCL, PCL), 1769 cm^−1^—Stretching vibr. of C=O (PLCL), 1722 cm^−1^—Stretching vibr. C=O (PCL), Figure S4: Graph of the measured values of fiber diameter from three independent series: cumulative PLCL/PCL blend, pure PCL and pure PLCL, Figure S5: FTIR analysis of the pure PCL, pure PLCL and spectra of PLCL/PCL blend from three separately electrospun materials.
