# Supplementary material for: The Effect of a Polyester Nanofibrous Membrane with a Fibrin-Platelet Lysate Coating on Keratinocytes and Endothelial Cells in a Co-Culture System
Source: Nanomaterials (Basel). 2021 Feb 11;11(2):457. doi: 10.3390/nano11020457 (PMC7916860; doi:10.3390/nano11020457)
Supplement: Supplementary file 1 [file nanomaterials-11-00457-s001.pdf]

nanomaterials

MDPI

Article

The Effect of a Polyester Nanofibrous Membrane with a Fibrin-Platelet Lysate Coating on Keratinocytes and Endothelial Cells in a Co-Culture System

Andreu Blanquer [1,*], Jana Musilkova [1], Elena Filova [1], Johanka Taborska [2], Eduard Brynda [2], Tomas Riedel [2], Andrea Klapstova [3], Vera Jencova [4], Jana Mullerova [4,5], Eva Kuzelova Kostakova [4], Renata Prochazkova [6,7] and Lucie Bacakova [1]

1 Institute of Physiology of the Czech Academy of Sciences, Videnska 1083, 142 20 Prague 4, Czech Republic; jana.musilkova@fgu.cas.cz (J.M.); elena.filova@fgu.cas.cz (E.F.); Lucie.Bacakova@fgu.cas.cz (L.B.)
2 Institute of Macromolecular Chemistry of the Czech Academy of Sciences, Heyrovskeho nam. 2, 162 06 Prague 6, Czech Republic; taborska@imc.cas.cz (J.T.); taborska@imc.cas.cz (E.B.); riedel@imc.cas.cz (T.R.)
3 Faculty of Textile Engineering, Technical University of Liberec, Studentska 1402/2, 461 17 Liberec 1, Czech Republic; andrea.klapstova@tul.cz
4 Faculty of Science, Humanities and Education, Technical University of Liberec, Studentska 1402/2, 461 17 Liberec 1, Czech Republic; vera.jencova@tul.cz (V.J.); Jana.mullerova@tul.cz (J.M.); Eva.kostakova@tul.cz (E.K.K.)
5 Institute of Nanomaterials, Advanced Technologies and Innovation, Bendlova 1409/7, 460 01 Liberec 1, Czech Republic
6 Faculty of Health, Technical University of Liberec, Studentska 1402/2, 461 17 Liberec 1, Czech Republic
7 Regional Hospital Liberec, Husova 357/28, 460 01 Liberec 1, Czech Republic; renata.prochazkova@nemlib.cz
* Correspondence: Andreu.blanquerjerez@fgu.cas.cz; Tel.: +420296443741

The effect of a polyester nanofibrous membrane with a fibrin-platelet lysate coating on keratinocytes and endothelial cells in a co-culture system

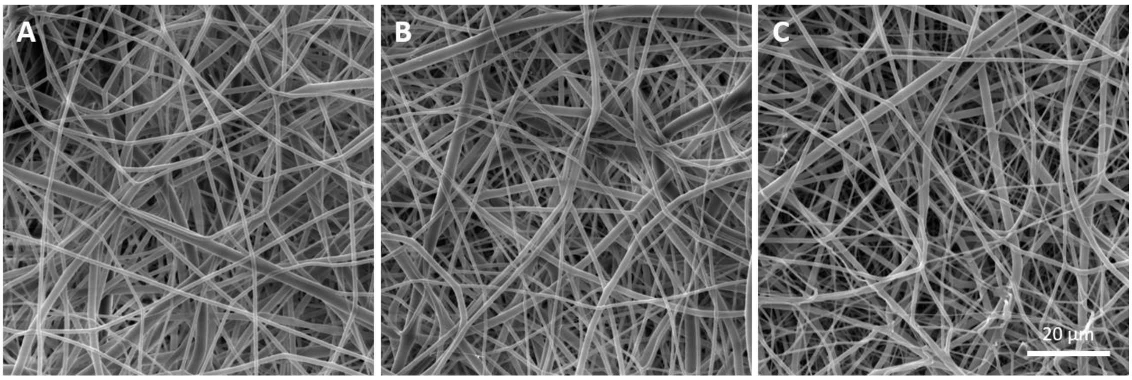

Figure S1. SEM images of the nanofibrous membranes: PLCL/PCL membrane (A), lyophilised PLCL/PCL membrane coated with fibrin (B) and lyophilised PLCL/PCL membrane coated with fibrin containing 50% of platelet lysate (C).

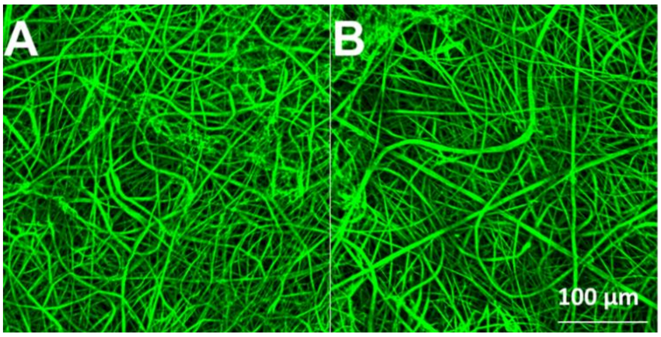

Figure S2. CLSM images of the fluorescence-stained proteins on the upper surface of the NF modified from a solution containing Fbg and 50% PL (NF50) observed in PBS after the modification (A) and after one week in PBS (B).

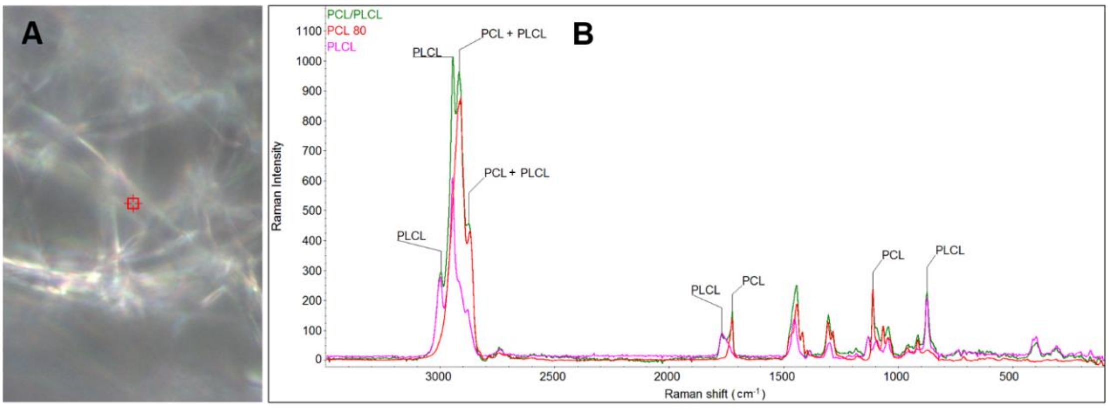

Figure S3. Raman spectroscopy analysis: (A) picture of analysed area, (B) obtained Raman shifts of pure PCL, pure PLCL and their PCL/PLCL blend: 3387 cm^{-1} – stretching vibr. of C-H (PLCL), 2944 cm^{-1} - stretching vibr. of C-H (PLCL), 2915 cm^{-1} - stretching vibr. of C-H (PLCL, PCL), 2873 cm^{-1} - stretching vibr. of C-H (PLCL, PCL), 1769 cm^{-1} – stretching vibr. of C=O (PLCL), 1722 cm^{-1} – stretching vibr. C=O (PCL).

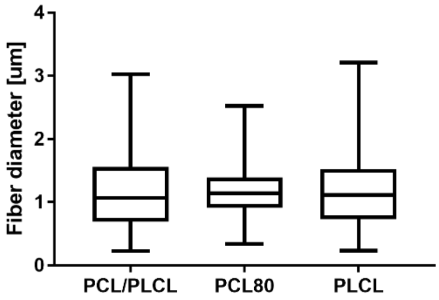

Figure S4. Graph of the measured values of fiber diameter from three independent series: cumulative PLCL/PCL blend, pure PCL and pure PLCL.

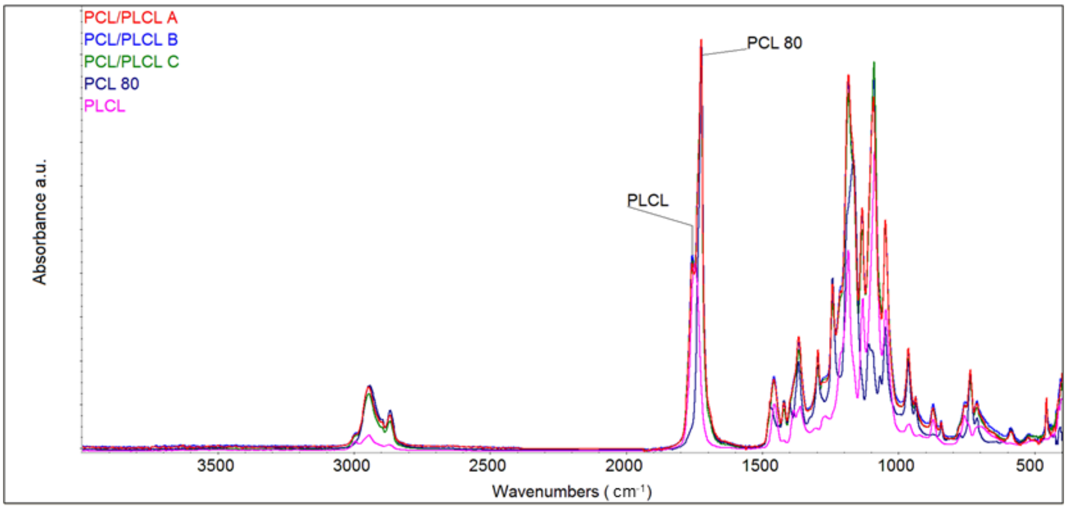

Figure S5. FTIR analysis of the pure PCL, pure PLCL and spectra of PLCL/PCL blend from three separately electrospun materials.
